# Conceptualization, Contexts, and Measurement of Nursing Theoretical Literacy: Protocol for a Scoping Review

**DOI:** 10.2196/92257

**Published:** 2026-05-27

**Authors:** Jiejie Ge, Haiyang Song, Jun Yang, Yanyu Feng, Ting Zheng, Min Wang, Min You, Sijing Peng

**Affiliations:** 1 School of Nursing, Anhui University of Chinese Medicine, Hefei, Anhui, China; 2 Department of Nursing, Huangshan City People’s Hospital, Huangshan, Anhui, China; 3 Department of Nursing, Second Affiliated Hospital of Anhui University of Chinese Medicine, Hefei, Anhui, China; 4 Key Laboratory of Geriatric Nursing and Health, School of Nursing, Anhui University of Chinese Medicine, Weishi Bldg, 4th Fl., 350 Longzihu St, Hefei, Anhui, 230012, China, 86 13675687458

**Keywords:** nursing theoretical literacy, nursing theory, theory-guided practice, theory-practice integration, competence, measurement, scoping review, protocol, artificial intelligence, AI

## Abstract

**Background:**

Nursing theory and conceptual models are central to nursing as a knowledge discipline, yet theory is often perceived as abstract and difficult to operationalize in education and practice. This theory-practice tension suggests variability in nurses’ and nursing students’ capability to access, interpret, critique, and apply theoretical knowledge in ways that shape praxis. Nursing theoretical literacy (NTL) is an emerging, practice-oriented literacy construct that may help specify this capability; however, its conceptual boundaries, contextual uses, and measurement approaches remain unclear.

**Objective:**

This scoping review aims to map how NTL and closely related constructs are conceptualized and defined in the nursing literature, characterize the contexts and populations in which they are discussed, applied, or studied, and assess the extent to which they have been operationalized and assessed.

**Methods:**

This scoping review will follow Joanna Briggs Institute guidance and be reported in accordance with the PRISMA-ScR (Preferred Reporting Items for Systematic Reviews and Meta-Analyses extension for Scoping Reviews). Using the population, concept, and context framework, we will include English- and Chinese-language sources across nursing education, clinical practice, leadership and management, and nursing research. We will search MEDLINE (PubMed), CINAHL, Embase, Scopus, Web of Science Core Collection, ERIC, China National Knowledge Infrastructure, Wanfang, VIP, and the Chinese Biomedical Database, complemented by gray literature searches of the Open Science Framework, dissertations and theses, institutional and professional association websites, and citation chasing. Two reviewers will independently screen titles, abstracts, and full texts, with disagreements resolved through consensus or by a third reviewer. Data will be charted using a piloted extraction form and synthesized through descriptive mapping, conceptual mapping of definitional elements and construct relationships, and a measurement inventory summarizing assessment approaches and reported measurement properties.

**Results:**

As of February 13, 2026, the PubMed and China National Knowledge Infrastructure search strategies have been pilot-tested and refined, confirming the feasibility of the concept mapping approach. Searches are planned for May to June 2026, screening is planned for June to July 2026, data charting is planned for July to August 2026, and synthesis and manuscript preparation are planned for August to September 2026. Results are expected to be submitted for publication in late 2026.

**Conclusions:**

This scoping review will provide an evidence map of how NTL is conceptualized, where it appears across nursing contexts, and how it is operationalized and assessed. Findings will describe how conceptual boundaries have been represented in the literature, identify gaps and priorities, and support downstream work, including hybrid concept analysis and the development or adaptation of NTL assessment tools.

## Introduction

Nursing is historically defined as a dual-natured discipline—simultaneously a rigorous knowledge domain and a high-stakes practice profession whereby nursing theories and conceptual models act as the primary engines of professional identity and clinical explanation. However, amid an increasingly digitalized and data-intensive health care landscape, nurses are expected to integrate theoretical reasoning with complex clinical decision-making and context-sensitive care. In many contemporary settings, nursing theory is frequently marginalized as abstract or secondary to empirically driven evidence-based practice and rigid algorithmic protocols. This systemic undervaluing persists despite evidence that theory-guided interventions such as those based on the caring theory by Watson [1] or embodied cognition significantly enhance clinical culture and patient safety [2,3]. The resulting praxis gap suggests that while practitioners may possess technical proficiency, they often lack the theoretical sensitivity required to ground complex clinical inquiry in nursing science [4,5].

In the context of global nursing scholarship, this challenge is particularly salient in regions transitioning from the importation of Western models to indigenous growth and localized theory development. Chinese scholars have recently emphasized that theoretical literacy is the most fundamental skill, serving as the critical juncture between professional cognition and behavioral competence [6,7]. According to recent educational frameworks, the improvement of theoretical literacy follows a scientific logic of profound understanding, internal identification, and practical integration [8]. This transition mirrors the mandate for competency-based education in the updated American Association of Colleges of Nursing Essentials, which requires graduates to synthesize complex evidence within a holistic theoretical framework [9].

Across the health professions, literacy constructs have increasingly been used to describe integrated capabilities that combine knowledge, skills, and dispositions. In nursing, related constructs such as genomic literacy and digital health literacy have helped shift attention from static knowledge acquisition toward competencies that can be supported, observed, and assessed in context. Within this broader landscape, nursing theoretical literacy (NTL) is used in this protocol as a provisional review label for a body of work concerned with nurses’ and nursing students’ engagement with nursing theories, conceptual models, and frameworks [10-12].

However, at this stage, NTL should not be treated as a fully settled construct with a single agreed-upon definition. Rather, the available literature suggests a potentially heterogeneous field in which the capacity to understand, critique, use, adapt, or integrate nursing theory may be described in different ways, across different contexts, and under different conceptual labels [10-13]. For this reason, the purpose of this scoping review is not to impose a final definition of NTL at the outset but to map how the literature itself conceptualizes and operationalizes this area of capability [14-16].

This need is further underscored by the rise of generative artificial intelligence (AI). As AI-enabled tools become more available in clinical and educational settings, nurses may face growing pressure to act efficiently while also preserving theoretically informed, context-sensitive, and ethically grounded judgment [17-19]. Therefore, a clear evidence map of how theory-related capability is described and assessed in nursing is needed. Against this background, this scoping review will examine how NTL and closely related constructs are defined; in which contexts they are discussed; and what instruments, indicators, or assessment approaches have been used or proposed to capture them [13,16,20,21].

This scoping review aims to systematically map the extent, nature, and characteristics of the literature relevant to NTL and closely related constructs. Consistent with the purpose of scoping reviews, the study begins from an open conceptual position and is designed to examine how the literature itself labels, defines, situates, and operationalizes this area of capability across nursing contexts [14,15]. Rather than assuming that NTL already has a fixed and agreed-upon conceptual boundary, this review seeks to clarify how the field currently describes and uses this concept and how such descriptions may support subsequent concept development and measurement work [22].

The review has 3 primary objectives. First, it will map how NTL and closely related constructs are conceptualized in the nursing literature, including the terminology used, the presence or absence of explicit definitions, and any proposed dimensions or conceptual features. Second, it will characterize the contexts and populations in which these constructs are discussed, including nursing education, clinical practice, leadership and management, and nursing research [21]. Third, it will identify and describe the assessment approaches used or proposed to capture NTL or related theory engagement capabilities, including instruments, rubrics, documentation audits, simulation-based indicators, and other assessment strategies, together with any reported information on their development and measurement properties [23].

In addition, the review will address several secondary objectives. These include examining adjacent constructs that may overlap conceptually with NTL; identifying reported antecedents and outcomes; mapping educational or organizational strategies intended to strengthen theory-related capability; and exploring cross-cultural and cross-linguistic variations, particularly between English- and Chinese-language literature [6-8,12,17,21].

Accordingly, the review is guided by the following primary questions:

1. How are NTL and closely related constructs conceptualized and defined in the nursing literature?
2. In which contexts, populations, and settings are these constructs discussed, applied, or studied?
3. What assessment approaches have been used or proposed to operationalize or evaluate NTL and related theory engagement capabilities?

The review will also address the following secondary questions:

1. What adjacent constructs are invoked in relation to NTL, and how do authors describe the relationships, overlaps, or distinctions between them [24-26]?
2. What antecedents, enabling conditions, and outcomes are reported or proposed in relation to NTL, including those relevant to technology-enabled nursing practice [17,18,27]?
3. What educational, organizational, or practice-based strategies are described as ways of strengthening NTL or related capabilities?
4. Are there discernible cross-cultural or cross-linguistic variations in how these constructs are described and assessed, particularly across English- and Chinese-language literature [6,8,21]?

## Methods

### Study Design and Reporting Standards

This scoping review will be conducted as a systematic evidence-mapping exercise following the updated Joanna Briggs Institute methodological guidance for scoping reviews [16]. The protocol is designed to provide a comprehensive interrogation of the evidence ecosystem surrounding NTL. Reporting will strictly adhere to the PRISMA-ScR (Preferred Reporting Items for Systematic Reviews and Meta-Analyses extension for Scoping Reviews) standards [28]. In line with *JMIR Research Protocols* requirements, a completed PRISMA-P (Preferred Reporting Items for Systematic Reviews and Meta-Analyses Protocols) checklist is provided as Checklist 1. The review will use an iterative approach: eligibility criteria and data charting items may be refined as the reviewers become familiar with the heterogeneous nature of the identified literature, with all such modifications documented as protocol amendments in the final report [15].

### Eligibility Criteria

Eligibility decisions will be governed by the population, concept, and context framework.

#### Population

We will include literature focusing on the entire nursing workforce spectrum: nursing students (all levels), registered nurses, advanced practice nurses, educators, preceptors, managers, and researchers. Studies involving mixed-professional groups (eg, physicians and nurses) will be included only if the nursing-specific findings are extractable or constitute the primary focus.

#### Concept

The focal concept for this review is NTL, which is treated as an emerging review construct rather than a fixed or fully standardized concept. For the purposes of this review, we will include literature that explicitly addresses the understanding, critique, use, application, or integration of nursing theories, conceptual models, or theoretical frameworks in nursing-related contexts. Because terminology in this field is likely to be inconsistent, we will also include closely related or proximate constructs when the source makes an explicit link to engagement with nursing theory, conceptual models, or frameworks. Examples may include theoretical competence, theory-guided practice, theory-based practice, and praxis-oriented theory application [6,8]. Sources focused solely on general critical thinking or evidence-based practice will be excluded unless they explicitly address nursing theory engagement.

For borderline cases, reviewers will judge whether the source contains an explicit and substantive link to nursing theory engagement. A source will be considered eligible when nursing theory, a nursing conceptual model, or a theoretical framework is used as part of the study aim, conceptual framing, intervention design, educational content, measurement approach, analysis, or interpretation. In contrast, sources will be excluded when theory is mentioned only as a general background term; when the focus is limited to general critical thinking, clinical reasoning, evidence-based practice, or professional competence without explicit engagement with nursing theory; or when the nursing-specific theoretical component cannot be separated from a broader interprofessional discussion. Borderline decisions will be documented during screening and resolved through discussion between 2 reviewers, with adjudication by a third reviewer when needed.

#### Context

No geographic or setting restrictions will be applied. We seek evidence from nursing education, clinical settings (including intelligent care environments), leadership, and research production [21].

#### Types of Evidence Sources

We will include empirical studies (quantitative, qualitative, and mixed methods), methodological papers, and conceptually oriented papers that contain explicit definitional, operational, or evaluative content relevant to NTL or closely related constructs. We will also include selected gray literature when it is likely to contain definitional, educational, organizational, or assessment-related material relevant to NTL, such as doctoral dissertations, institutional training documents, and professional association publications. Conference abstracts without sufficient detail for conceptual or methodological interpretation will not be included.

#### Language and Time Frame

English- and Chinese-language sources will be included. No restrictions will be applied to publication year. Sources available from database inception to the final search date will be considered eligible provided that they meet the population, concept, and context criteria and other eligibility requirements.

### Information Sources and Search Strategy

A three-step structured search strategy will be executed to maximize recall:

1. Initial limited search: conducted in PubMed and China National Knowledge Infrastructure (CNKI) to identify keywords and indexing terms from high-relevance papers.
2. Comprehensive database search: this will span MEDLINE (PubMed), CINAHL, Embase, Scopus, Web of Science, ERIC, and Chinese repositories (CNKI, Wanfang, VIP, and the Chinese Biomedical Database).
3. Citation chasing: we will use Citationchaser for forward and backward tracking and manually screen the reference lists of key reviews [20].

The search strategy will follow a structured, multistep approach recommended for scoping reviews, beginning with an initial limited search to refine keywords and indexing terms followed by comprehensive database searches and then citation-based searching. Search terms will combine (1) NTL-specific and theory literacy phrases; (2) nursing theory, model, or framework terminology; and (3) capability or competence and application terms such as “theory use,” “theory-guided,” “conceptual model,” “framework,” “competence,” “literacy,” and “integration.” Chinese keywords will be developed in parallel to capture common translations and local use, such as “护理理论素养/理论素养,” “理论运用/理论指导实践,” “理论-实践整合,” and “概念模型/理论框架,” and adapted based on database indexing and retrieval characteristics. A health sciences librarian will be consulted to audit the Boolean logic and term mapping, such as mapping “praxis” to “理论实践整合” in Chinese contexts. Full search strings for PubMed and CNKI are available in Multimedia Appendix 1 and Multimedia Appendix 2, respectively.

### Study Selection Process and Reliability Procedures

**Figure 1.**
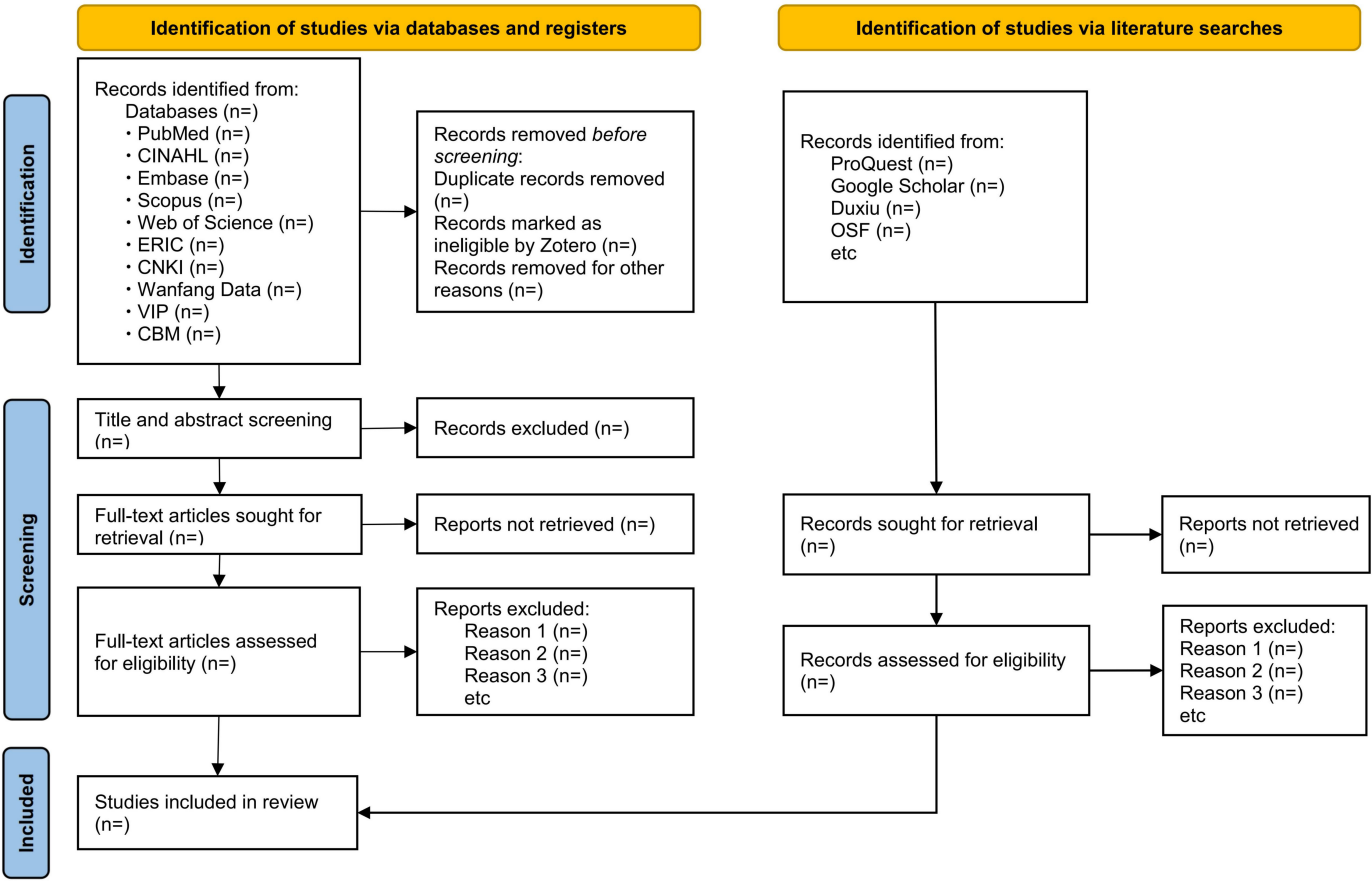
PRISMA (Preferred Reporting Items for Systematic Reviews and Meta-Analyses) flow diagram. CNKI: China National Knowledge Infrastructure; OSF: Open Science Framework.

All records will be exported into a reference manager software for deduplication and then imported into a systematic review management platform such as Covidence or Rayyan for screening. Two reviewers will independently screen titles, abstracts, and full texts against the eligibility criteria. Disagreements will be resolved through discussion; if consensus cannot be reached, a third reviewer will adjudicate. A pilot screening exercise will be conducted on a purposive sample of 50 citation records to test the clarity of the eligibility criteria and the inclusion and exclusion decision rules (Multimedia Appendix 3). Interreviewer agreement will be monitored during piloting, primarily as a process control indicator rather than as a quality metric. The study selection process will ultimately be summarized in a PRISMA (Preferred Reporting Items for Systematic Reviews and Meta-Analyses) 2020 flow diagram, which is shown in Figure 1 [20].

### Data Extraction (Charting) and Data Management

A standardized data extraction form will be developed and piloted and then applied by 2 reviewers (independently or in duplicate with verification depending on volume). Pilot charting will be conducted on a purposive sample of approximately 5 to 10 included sources to refine the standardized data charting form (Multimedia Appendix 4). Extracted data will be managed in Microsoft Excel, with version control and an audit trail for changes.

Data items will be designed to directly support the primary aims (conceptualization, contexts, and measurement) while enabling secondary mapping (construct ecosystem, reported linkages, and strategies). Extracted fields will include bibliographic details; country or region; study type and design; participant group; setting and context category; terms used to represent NTL or related constructs; presence and content of explicit definitions; implicit conceptual features and proposed dimensions; adjacent constructs invoked and stated relationships or boundary claims; operationalization approaches, such as assignments, documentation artifacts, and competency frameworks; measurement tools or approaches, such as instrument name, domains, item sources if reported, scoring, feasibility and administration, language versions, availability, and any reported reliability or validity evidence; and any author-reported antecedents and enablers, outcomes, and proposed pathways. When intervention or strategy content is present, we will extract intervention characteristics (educational and organizational); target level (learner, clinician, unit, and system); and reported implementation considerations, such as acceptability, feasibility, fidelity, and outcomes. The standardized data charting form and extraction fields can be found in Multimedia Appendix 4.

### Critical Appraisal of Evidence

Consistent with the purpose of a scoping review, formal risk-of-bias appraisal will not be undertaken. The aim of this review is to map the available literature and characterize how NTL and related constructs have been conceptualized, contextualized, and assessed rather than determine the comparative methodological quality of individual studies or instruments. Where assessment tools or measurement approaches are identified, any reported psychometric information, such as reliability, validity, development procedures, or feasibility data, will be extracted and presented descriptively. These data will be used to characterize the measurement landscape, not to provide a formal quality rating or comparative judgment regarding instrument robustness.

### Data Synthesis and Presentation of Results

Synthesis will use a trilayered mapping approach:

1. Descriptive synthesis: frequencies of publication types, populations, and settings, visualized through heat maps or context-by-population matrices
2. Conceptual mapping: thematic synthesis of NTL attributes and a network visualization of the construct ecosystem documenting the relationship between NTL and adjacent terms such as “theoretical sensitivity” [25,26]
3. Measurement inventory: a catalog of existing tools stratified by target competency level and clinical domain (eg, surgical vs geriatric care) [3,11]

Planned reporting templates for presenting the mapped conceptualizations, contexts, and measurement evidence are provided in Multimedia Appendix 5.

### Protocol Registration and Amendments

This protocol was registered on the Open Science Framework (OSF; registration number: RJ5AU). Any protocol amendments such as refinement of eligibility criteria, expansion of information sources, or modification of charting items during pilot procedures will be documented with rationale and time-stamped in the OSF record and transparently reported in the completed scoping review.

### Patient and Public Involvement

No patients or members of the public will be involved in the design or conduct of this scoping review.

### Ethical Considerations

Ethics approval is not required because the review involves analysis of publicly available literature and does not involve human participant recruitment or identifiable private data. Any included gray literature will be handled in accordance with applicable copyright and access conditions, and sources will be cited appropriately.

## Results

As of February 13, 2026, this review is in the preparatory phase. The protocol has been registered on the OSF, and the PubMed and CNKI search strategies have been pilot-tested and refined. Pilot-testing indicated that the search strategies retrieve benchmark records representing both English-language literacy framings and commonly used Chinese terms (eg, “lilun suyang” meaning theoretical literacy and “lilun suzhi” meaning theoretical quality or theoretical competence or capacity) relevant to NTL [8].

We anticipate that the searches will be conducted from May to June 2026, followed by title and abstract screening from June to July 2026, full-text retrieval and data charting from July to August 2026, and synthesis and manuscript preparation from August to September 2026. The finalized evidence map is expected to be submitted for peer-reviewed publication in late 2026 [16].

The review is expected to produce (1) a descriptive matrix of NTL-related applications across global nursing education and intelligent care environments [21], (2) a conceptual network visualization showing how relationships between NTL and proximate constructs such as clinical reasoning and praxis have been described in the literature, and (3) a stratified measurement inventory of existing tools and assessment approaches relevant to NTL-related capabilities [23].

## Discussion

### Principal Results

This scoping review is designed to map the fragmented literature surrounding NTL and related theory engagement constructs. By examining the terminology, definitions, contexts, and assessment approaches reported in the literature, the review will describe how NTL has been represented and distinguished from adjacent constructs rather than establish a definitive conceptual boundary. In this sense, the review is intended to provide an evidence map that can inform subsequent concept analysis, theoretical refinement, and measurement development.

As generative AI becomes increasingly embedded in education and health care, nurses may need to preserve theoretically informed and context-sensitive judgment while working with digital and algorithmic tools. Without sufficient capacity to interpret phenomena through nursing science, practitioners may risk cognitive offloading, whereby reliance on machine-generated outputs weakens humanistic and theoretical reasoning at the core of professional identity [8,12,19]. By mapping how NTL and related constructs have been discussed, this review will provide a foundation for future research on theory-informed nursing practice in technologically changing environments.

### Global and Cross-Cultural Significance

A unique strength of this review is its explicit intent to bridge divergent knowledge traditions. By synthesizing both English- and Chinese-language scholarship, we aim to uncover how NTL manifests in regions transitioning from theoretical importation to indigenous theory growth [6]. This dual perspective may inform future measurement work by highlighting the need for assessment approaches that are culturally sensitive and contextually relevant. The inclusion of gray literature such as institutional training manuals and nursing management innovation frameworks may also help identify how theory-related capability is represented in routine educational, managerial, or practice-oriented documents [21].

### Limitations

We acknowledge several methodological challenges. First, the terminological fluidity surrounding literacy, competence, theory use, and related constructs may reduce search precision and increase screening complexity. To mitigate this risk, we will use pilot screening to calibrate decision rules and monitor agreement as process indicators, with discrepancies resolved through discussion and adjudication. Second, consistent with scoping review conventions, formal risk-of-bias assessment will not be undertaken, which limits the extent to which the review can support evaluative judgments about evidence quality. To address this limitation, we will extract and present any reported psychometric information descriptively to characterize rather than formally rate the measurement landscape. Finally, this protocol does not include a formal expert consultation phase. Although such consultation is not essential for this scoping review, it may represent an important next step in subsequent phases of the broader research program, particularly for concept refinement and future instrument development.

### Conclusions

Ultimately, the NTL evidence map will provide a structured basis for subsequent research. Rather than offering a final definition of theoretical literacy, the review will summarize how theory-related capability has been described, situated, and assessed across nursing contexts. These findings may inform subsequent concept analysis; theoretical refinement; and the development or adaptation of assessment approaches aligned with contemporary competency frameworks, including the updated American Association of Colleges of Nursing Essentials [9]. Therefore, strengthening attention to NTL is relevant not only to nursing scholarship but also to the preparation of a workforce capable of maintaining theoretically informed and ethically grounded practice in an AI-enhanced health care environment.

## Supplementary material

10.2196/92257 Multimedia Appendix 1 Full search strategy for PubMed (MEDLINE).

10.2196/92257 Multimedia Appendix 2 Full search strategy for China National Knowledge Infrastructure.

10.2196/92257 Multimedia Appendix 3 Screening decision rules and exclusion reason codes.

10.2196/92257 Multimedia Appendix 4 Standardized data charting form (extraction fields).

10.2196/92257 Multimedia Appendix 5 Planned reporting templates.

10.2196/92257 Checklist 1 PRISMA-P checklist.

## Abbreviations

AI: artificial intelligence
CNKI: China National Knowledge Infrastructure
NTL: nursing theoretical literacy
OSF: Open Science Framework
PRISMA: Preferred Reporting Items for Systematic Reviews and Meta-Analyses
PRISMA-P: Preferred Reporting Items for Systematic Reviews and Meta-Analyses Protocols
PRISMA-ScR: Preferred Reporting Items for Systematic Reviews and Meta-Analyses extension for Scoping Reviews
