# Supplementary material for: Conceptualization, Contexts, and Measurement of Nursing Theoretical Literacy: Protocol for a Scoping Review
Source: JMIR Res Protoc. 2026 May 27;15:e92257. doi: 10.2196/92257 (PMC13215574; doi:10.2196/92257)
Supplement: Multimedia Appendix 1 [file resprot-v15-e92257-s001.docx]

Database: PubMed (MEDLINE)

Planned final run date: February 2026 (pilot-tested on February 13, 2026)

| #Step | Search Terms and Boolean Logic |
| --- | --- |
| #1 | ("Nursing"[Mesh] OR "Nurses"[Mesh] OR "Students, Nursing"[Mesh] OR nurs*[tiab] OR nurse*[tiab]) |
| #2 | ("nursing theoretical literacy"[tiab] OR "theoretical literacy"[tiab] OR "theory literacy"[tiab] OR "theoretical literac*"[tiab] OR "theory literac*"[tiab] OR "theoretical competenc*"[tiab] OR "theory competenc*"[tiab] OR "theoretical capability"[tiab] OR "theory capability"[tiab] OR "theory-based practice competenc*"[tiab] OR "theory guided practice competenc*"[tiab] OR "theory-guided practice competenc*"[tiab]) |
| #3 | ("Nursing Theory"[Mesh] OR "Models, Nursing"[Mesh] OR "nursing theor*"[tiab] OR "nursing model*"[tiab] OR "conceptual model*"[tiab] OR "conceptual framework*"[tiab] OR framework*[tiab]) |
| #4 | ("theory use"[tiab] OR "use of theory"[tiab] OR "theory-guided"[tiab] OR "theory guided"[tiab] OR "theory-based"[tiab] OR "theory based"[tiab] OR "theory-informed"[tiab] OR "theory informed"[tiab] OR "theory application"[tiab] OR "theory utilization"[tiab] OR "theory utilisation"[tiab] OR "theory-practice integration"[tiab] OR "theory-practice integration"[tiab] OR "theory practice integration"[tiab] OR "theory-practice gap"[tiab] OR "theory-practice gap"[tiab] OR "theory practice gap"[tiab]) |
| #5 | #2 OR (#3 AND #4) |
| #6 | #1 AND #5 |
| #7 | #6 NOT (animals[mh] NOT humans[mh]) |
