# Supplementary material for: Conceptualization, Contexts, and Measurement of Nursing Theoretical Literacy: Protocol for a Scoping Review
Source: JMIR Res Protoc. 2026 May 27;15:e92257. doi: 10.2196/92257 (PMC13215574; doi:10.2196/92257)
Supplement: Multimedia Appendix 2 [file resprot-v15-e92257-s002.docx]

Database: CNKI (China National Knowledge Infrastructure)

Collections: CNKI Journal (CJFD); Master’s & Doctoral Dissertations; Conference Proceedings (as available)

Field: Subject/Theme (主题)

Time span: Inception to February 2026 (no start-date restriction)

Planned final run date: February 2026 (pilot-tested on February 13, 2026)

| #Step | Search Terms and Boolean Logic |
| --- | --- |
| #1 | SU=护理 OR 护士 OR 护理人员 OR 护理学生 OR 护理教育 OR 护理教师 OR 护理管理 OR 护理科研 |
| #2 | SU=护理理论 OR 护理学理论 OR 护理理论学 OR 护理模型 OR 护理模式 OR 概念模型 OR 理论框架 OR 概念框架 |
| #3 | SU=护理理论素养 OR 理论素养 OR 理论能力 OR 理论素质 OR 理论水平 OR 理论运用 OR 理论应用 OR 理论使用 OR 理论指导 OR 理论指导实践 OR 理论导向 OR 理论引导 OR 理论实践整合 OR 理论-实践整合 OR 理论与实践整合 |
| #4 | #2 AND #3 |
| #5 | #1 AND #4) |

The final CNKI query will be executed using CNKI Advanced Search; any database-specific syntax adjustments will be recorded in the OSF log.
