# Supplementary material for: Conceptualization, Contexts, and Measurement of Nursing Theoretical Literacy: Protocol for a Scoping Review
Source: JMIR Res Protoc. 2026 May 27;15:e92257. doi: 10.2196/92257 (PMC13215574; doi:10.2196/92257)
Supplement: Multimedia Appendix 3 [file resprot-v15-e92257-s003.docx]

Purpose: To standardize eligibility decisions and minimize drift during title/abstract and full-text screening for the Nursing Theoretical Literacy (NTL) scoping review. Codes are applied at the first screening stage where the exclusion is justified.

General decision rules: (1) Include sources that explicitly conceptualize, operationalize, or measure NTL or proximate constructs (eg, theoretical competence, theory literacy) in nursing. (2) Include sources discussing nursing theories/models/frameworks if they address the capability, disposition, or competence to understand, critique, select, apply, or integrate theory in nursing education, practice, management, or research. (3) Adjacent constructs (eg, evidence-based practice, critical thinking) are included only when the source makes an explicit conceptual link to theory engagement or uses them as boundaries/antecedents/outcomes of NTL. (4) For 'theory-practice gap' sources, include only if the discussion is anchored in nursing theories/models/frameworks or in nurses’/students’ ability to work with such theories in practice.

| **Code** | **Exclusion reason** | **Operational definition** | **Examples / notes** |
| --- | --- | --- | --- |
| E01 | Not nursing-focused | Primary topic is not nursing (profession, students, education, practice, management, or nursing research). | Allied health theory education without nursing subgroup. |
| E02 | No nursing theory/model/framework component | Does not involve nursing theories, nursing conceptual models, or explicitly nursing-relevant conceptual frameworks. | Generic 'theory' only; business/engineering frameworks. |
| E03 | No theory engagement capability/literacy/competence construct | Mentions nursing theory but does not address abilities, skills, dispositions, literacy, competence, or capacity to use /understand /critique /apply theory. | History/summary of a nursing model. |
| E04 | Different target construct (not proximate) | Focus is not NTL or a proximate construct and is not positioned as conceptually related (boundary/indicator/antecedent/outcome) to NTL. | General critical thinking with no theory content/link. |
| E05 | Non-empirical and irrelevant | Brief news item or opinion without substantive conceptual, methodological, or measurement content relevant to NTL aims. | Editorials may be included if they define/argue for theory literacy. |
| E06 | Outside scope population/context | Population/context is outside the nursing contexts defined by the protocol’s PCC. | Medical students only; non-nursing organizations. |
| E07 | Non-retrievable full text | Full text cannot be obtained after documented reasonable attempts. | Record requests, dates, and sources searched. |
| E08 | Duplicate/overlap | Duplicate record or overlapping publication reporting identical content. | Keep the most complete /peer-reviewed version. |
| E09 | Non-human/animal-only | Animal/biomedical studies with no human nursing theory/literacy content. | Rare; usually irrelevant. |

Reliability plan: Pilot screening will be conducted on an initial subset (eg, 50 records) to calibrate reviewers and refine exclusion codes. Inter-reviewer agreement will be monitored (eg, percent agreement and/or Cohen’s kappa as process indicators), and discrepancies will be reconciled through discussion or adjudication by a third reviewer, with decision rules logged as protocol amendments.
