# Supplementary material for: Conceptualization, Contexts, and Measurement of Nursing Theoretical Literacy: Protocol for a Scoping Review
Source: JMIR Res Protoc. 2026 May 27;15:e92257. doi: 10.2196/92257 (PMC13215574; doi:10.2196/92257)
Supplement: Multimedia Appendix 4 [file resprot-v15-e92257-s004.docx]

| **Domain** | **Extraction field** | **Operational definition / coding guidance** | **Data type / allowable values**  **(examples)** | **Notes** |
| --- | --- | --- | --- | --- |
| Bibliographic & administrative | Unique ID | Internal ID assigned by review team for traceability. | String (e.g., NTL_0001) |  |
| Bibliographic & administrative | Full citation | Author(s), year, title, source. | Text |  |
| Bibliographic & administrative | DOI/URL | Record DOI; if not available, record stable URL. | Text |  |
| Bibliographic & administrative | Country/region | Country/region of study or originating organization (if conceptual/guidance). | Text | If multi-country, record all. |
| Bibliographic & administrative | Language | Language of full text. | English / Chinese / Other |  |
| Bibliographic & administrative | Publication year | Year of publication (or release). | YYYY |  |
| Bibliographic & administrative | Source type | Classify evidence source. | Empirical-quant / Empirical-qual / Mixed-methods / Conceptual-theoretical / Methodological / Review / Grey literature (thesis, curriculum, guideline, report) |  |
| PCC mapping | Population group(s) | Primary target group(s) addressed. | Students / Registered nurses / APRN / Educators / Leaders-managers / Researchers / Mixed | If mixed, specify nursing subgroup. |
| PCC mapping | Setting / sector | Where the work is situated. | Education / Clinical practice / Leadership-management / Research / Multi-context |  |
| PCC mapping | Context details | Free-text description of setting (e.g., undergraduate program, ICU, hospital education dept.). | Text |  |
| Study characteristics (empirical only) | Design | Study design as reported. | Cross-sectional / Experimental / Qualitative interview / Delphi / Psychometric validation / etc. | Not applicable to purely conceptual sources. |
| Study characteristics (empirical only) | Sample size | Number of participants/sources. | Integer | Not reported if absent. |
| Study characteristics (empirical only) | Participant characteristics | Key demographics/professional level as reported. | Text |  |
| Terminology | Index construct label(s) | Term(s) used by authors to name the focal construct (verbatim). | Text | Retain original wording. |
| Terminology | NTL-labelled vs adjacent-labelled | Code whether authors explicitly label as NTL (or direct equivalent) vs an adjacent construct. | NTL-labelled / Adjacent-labelled | Decide based on authors’ wording. |
| Conceptualization | Explicit definition present? | Whether an explicit definition statement is provided. | Yes / No |  |
| Conceptualization | Definition excerpt | Short excerpt capturing the definition (avoid long copyrighted text). | Text (≤1–2 sentences) | If needed, paraphrase and note. |
| Conceptualization | Implicit conceptual features | Key elements implied (e.g., access, interpret, critique, apply theory). | Coded list + supporting notes | Use a controlled codebook. |
| Conceptualization | Proposed domains/dimensions | Any dimensional structure proposed/used. | Text + coded categories | Record names of domains. |
| Construct ecosystem & boundaries | Adjacent constructs invoked | List constructs discussed as related/overlapping (e.g., theory use, theory-guided practice). | Text (list) |  |
| Construct ecosystem & boundaries | Boundary/relationship claim type | How authors position NTL vs adjacent constructs. | Overlap / Subset / Distinct / Synonymous / Unclear | Per adjacent construct if possible. |
| Operationalization | Operationalization approach | How the construct is enacted/assessed (e.g., assignments, documentation audits). | Text |  |
| Measurement inventory | Assessment approach type | Classify approach. | Scale / Rubric / Audit indicator / Observation checklist / Competency framework / Other |  |
| Measurement inventory | Instrument/tool name | Name and citation of instrument/rubric/tool. | Text |  |
| Measurement inventory | Target population/context | Population and context where tool is used. | Text |  |
| Measurement inventory | Domains/subscales | Domains/subscales as reported. | Text |  |
| Measurement inventory | Scoring & administration | Scoring method, respondent, mode, time burden if reported. | Text | Not reported if absent. |
| Measurement inventory | Availability | Whether items/tool are accessible. | Open / Partially available / Not available / Unclear |  |
| Measurement evidence | Reliability evidence | Any reliability evidence reported (e.g., alpha, test-retest). | Text | Extract statistics if present. |
| Measurement evidence | Validity evidence | Any validity evidence reported (content/construct/criterion). | Text |  |
| Measurement evidence | Feasibility/acceptability | Any feasibility data or stakeholder feedback. | Text |  |
| Reported linkages | Antecedents/enablers | Author-reported or proposed antecedents/enabling conditions. | Text (coded list + notes) |  |
| Reported linkages | Outcomes (proximal) | Observable proximal outcomes attributed to NTL. | Text (coded list + notes) |  |
| Reported linkages | Outcomes (distal) | Broader downstream outcomes (if described). | Text |  |
| Reported linkages | Proposed pathways/mechanisms | Any proposed pathways/linkages (descriptive). | Text |  |
| Strategies/interventions | Strategy present? | Whether any strategy/intervention aligned with NTL is described. | Yes / No |  |
| Strategies/interventions | Strategy description | Components, delivery mode, duration, dose, setting. | Text |  |
| Strategies/interventions | Implementation considerations | Acceptability, feasibility, fidelity, barriers/facilitators. | Text |  |
| Strategies/interventions | Reported outcomes/metrics | Outcomes used to evaluate strategy (if any). | Text |  |
| Reviewer notes | Inclusion rationale | Brief rationale for inclusion if borderline. | Text |  |
| Reviewer notes | Extraction notes | Ambiguities, assumptions, follow-up needed (e.g., contact authors). | Text |  |

**Coding note.** Controlled vocabularies should be finalized during pilot charting. Any field refinements will be versioned and timestamped in OSF.
