# Supplementary material for: Conceptualization, Contexts, and Measurement of Nursing Theoretical Literacy: Protocol for a Scoping Review
Source: JMIR Res Protoc. 2026 May 27;15:e92257. doi: 10.2196/92257 (PMC13215574; doi:10.2196/92257)
Supplement: Multimedia Appendix 5 [file resprot-v15-e92257-s005.docx]

***Note:*** This appendix provides reporting templates for the final scoping review manuscript. They are included here to increase transparency and reduce analytic flexibility. These tables will be populated after screening and data charting are completed.

Template A. Characteristics of included sources (skeleton).

| **Study ID** | **Citation** | **Country/region** | **Language** | **Source type** | **Design/method** | **Population** | **Setting/context** | **Key theory/model referenced** |
| --- | --- | --- | --- | --- | --- | --- | --- | --- |
| — | — | — | — | — | — | — | — | — |

Template B. Conceptualization map (how NTL is defined and structured).

| **Study ID** | **Index term**  **(s)** | **Explicit definition**  **(verbatim)** | **Key attributes/dimensions** | **Boundary claims vs adjacent constructs** | **Antecedents** | **Outcomes/impacts** |
| --- | --- | --- | --- | --- | --- | --- |
| — | — | — | — | — | — | — |

Template C. Measurement inventory (if instruments/tools are identified).

| **Instrument/tool** | **Construct label** | **Domains/subscales** | **Items**  **(n)** | **Response format** | **Scoring** | **Target population** | **Context** | **Psychometric evidence reported** | **Accessibility** |
| --- | --- | --- | --- | --- | --- | --- | --- | --- | --- |
| — | — | — | — | — | — | — | — | — | — |
